# Association of Visual, Hearing, and Dual Sensory Impairment With Incident Dementia

**DOI:** 10.3389/fnagi.2022.872967

**Published:** 2022-06-14

**Authors:** Wenyi Hu, Yueye Wang, Wei Wang, Xinyu Zhang, Xianwen Shang, Huan Liao, Yifan Chen, Yu Huang, Xueli Zhang, Shulin Tang, Honghua Yu, Xiaohong Yang, Mingguang He, Zhuoting Zhu

**Affiliations:** ^1^Department of Ophthalmology, Guangdong Academy of Medical Sciences, Guangdong Provincial People's Hospital, Guangzhou, China; ^2^Centre for Eye Research Australia, Ophthalmology, University of Melbourne, Melbourne, VIC, Australia; ^3^Ophthalmology, Department of Surgery, University of Melbourne, Melbourne, VIC, Australia; ^4^State Key Laboratory of Ophthalmology, Zhongshan Ophthalmic Center, Sun Yat-sen University, Guangzhou, China; ^5^Shanghai Jiaotong University School of Medicine, Shanghai, China; ^6^Neural Regeneration Group, Institute of Reconstructive Neurobiology, University of Bonn, Bonn, Germany; ^7^John Radcliffe Hospital, Oxford University Hospitals NHS Foundation Trust, Oxford, United Kingdom

**Keywords:** dual sensory impairment (DSI), dementia, prevention, visual impairment (VI), hearing impairment (HI)

## Abstract

**Introduction:**

The relationship between sensory impairments and the risk of dementia is inconclusive. We aim to investigate the association of visual impairment (VI), hearing impairment (HI), and dual sensory impairment (DSI) with incident dementia.

**Methods:**

The UK Biobank study recruited more than 500,000 participants aged 40–69 years across the United Kingdom. Participants with available visual acuity (VA) measurements and speech-reception-threshold (SRT) information and free of dementia at the baseline assessment were included in the analysis. VI was defined as VA worse than 0.3 LogMAR units and HI were defined as an SRT of −5.5 dB or over. DSI was defined as the presence of both VI and HI. Incident dementia was identified through linked data to primary care or hospital admission records and death registries. Multivariable Cox proportional hazard regression models were used to examine the association of VI, HI, and DSI with incident dementia.

**Results:**

Among 113,511 participants (mean age: 56.8 ± 8.09 years, female: 54.4%), a total number of 1,135 (1.00%) cases of incident dementia were identified during a median follow up period of 11.1 years [interquartile range (IQR): 10.9–11.4 years]. The incidence of dementia showed significant differences among the non-sensory impairment (NSI) group, VI-only group, HI-only group, and DSI group (*p* < 0.001). After adjusting for demographic, lifestyle, health, and genetic factors, isolated VI (*HR* = 1.50, 95% *CI*: 1.06–2.12, *p* = 0.023), isolated HI (*HR* = 1.42, 95% *CI*:1.20–1.69, *p* < 0.001), and DSI (*HR* = 1.82, 95% *CI*: 1.10–3.00, *p* = 0.020) were independently associated with higher risks of incident dementia.

**Conclusions:**

Visual, hearing, and dual sensory impairments were associated with an increased risk of developing dementia, suggesting that visual and hearing impairments are modifiable risk factors that can be targeted to prevent dementia.

## Introduction

Dementia was estimated to affect 50 million people worldwide in 2018, and the number has been projected to triple by 2050 (Patterson, 2018). The annual cost of dementia was ~1 trillion US dollars globally (Patterson, 2018). Driven by the growth of population and the increasing life expectancy, the disease burden is expected to continue to increase (Alzheimer's Association, 2020). With limited treatment available, identifying modifiable risk factors as potential targets for intervention is critical for reducing the burden of dementia (Livingston et al., 2017, 2020).

Of note, sensory impairments, which are common in the elderly population (Campbell et al., 1999; Swenor et al., 2013), have been implicated in the development of dementia (Lin et al., 2011; Shang et al., 2021). Mounting evidence has shown that visual impairment (VI) and hearing impairment (HI) were independently associated with dementia and cognitive impairment (Uhlmann et al., 1991; Lin et al., 2011, 2013; Gurgel et al., 2014; Loughrey et al., 2018; Liu and Lee, 2019; Michalowsky et al., 2019; Lee et al., 2020; Tran et al., 2020; Chen et al., 2021), which might be explained by theories of sensory deprivation, information input degradation, and cognitive load (Yamada et al., 2016). This led to further investigations of the differential effects of VI, HI, and dual sensory impairment (DSI) on dementia (Lin et al., 2004; Hong et al., 2016; Brenowitz et al., 2019). However, there was significant heterogeneity in the methodology including the study population selected, measurements of sensory impairments, follow-up period and definition of cognitive function, and consequently inconsistent results were reported. In particular, whether subjective or objective measurements of VI and HI were performed, and the discrepancy in the sensitivity of the sensory tests could result in different findings for the association found between sensory impairments and dementia (Luo et al., 2018; Michalowsky et al., 2019; Hwang et al., 2020; Byeon et al., 2021). Further studies are warranted to examine the association of single and DSI with incident dementia.

Therefore, we aim to investigate the association of VI, HI, and DSI with incident dementia in the large community-dwelling population of the UK Biobank study.

## Methods

### Study Population

From March 13, 2006 to December 1, 2010, the UK Biobank recruited and received informed consent from 502,462 participants across the United Kingdom in 22 centers in England, Scotland, and Wales. The detailed study protocol was described elsewhere (Sudlow et al., 2015). In brief, for collection of baseline characteristics, each participant completed touch-screen questionnaires, underwent physical measurements, and provided biological samples (Sudlow et al., 2015). Visual (Chua et al., 2019) and hearing (Dawes et al., 2014) measurements commenced in some of the 22 centers in late 2009, and visual acuity (VA) data and speech-reception-threshold (SRT) scores were collected from 117,715 to 164,770 participants, respectively. Individuals with at least one side or both sides of available data of VA and SRT information were included, and those with history of dementia at baseline were excluded. 113,511 participants in total were included in the study. For each variable, missing or substandard (e.g., those recorded as reluctant to answer) values were discarded.

The UK Biobank Study received ethical approval from North West Multi-Centre Research Ethics Committee (11/NW/0382). Access to the data was granted through application and the application number for the present study is 62,525. The written informed consent was obtained from all the participants of this study. The study was conducted adhering to the tenets of the Declaration of Helsinki.

### Sensory Impairment

Visual impairment was determined based on “better-seeing eye” performance based on the lower logarithm of the minimum angle of resolution (LogMAR) value of either left (UKB Field 5208-0.0) or right (UKB Field 5201-0.0) eye. Briefly, participants read letters on the LogMAR chart from the top in a size sequence till they identified 2 letters incorrectly at a distance of 4 meters or 1 meter, if they could not read. Presenting VA was measured, which is with optical correction, if any, the participant is currently using. The number of correctly identified letters was converted to LogMAR VA, and VI was defined as VA worse than 0.3 LogMAR units (Snellen 20/40).

Hearing impairment was measured based on “better-ear” performance, namely the lower SRT value of either left (UKB Field 20019-0.0) or right (UKB Field 20021-0.0) ear. The SRT was estimated through the Digit Triplet Test, which, in brief, is an automated hearing test on how well the participant can hear three spoken numbers (a triplet, i.e., signal) played with a speech-shaped noise in the background. Throughout the test, the signal-to-noise ratio (SNR) was defined as half of the presented speech can be understood correctly, and SRT was the SNR measured in the last round of all 15 test rounds. Therefore, those with SRT of −5.5 dB or over were determined as HI. Dual sensory impairment was defined when one possessed both VI and HI.

### Ascertainment of Dementia

The objective outcome was the incidence of dementia, which is defined as dementia that is censored after the date of baseline assessment (UKB Field 53-0.0). The International Classification of Diseases (ICD) was used to identify cases with all-cause dementia, both ICD-9 and ICD-10 codes were used. Database for the ascertainment of dementia included self-reported data (participants indicated a history of dementia diagnosed by a doctor during the baseline nurse-led interview were excluded from analysis), primary care or hospital records of dementia, or cause of death in NHS Information Centre and the NHS Central Register Scotland. The follow-up period was calculated from the baseline to the first occurrence of incident dementia, death, loss to follow-up or the last follow-up date (April 28, 2021), whichever came first.

### Covariates

Age (UKB Field 21022-0.0) and gender (UKB Field 31-0.0) were original values collected from the touch-screen system. Ethnicity (UKB Field 21000-0.0) were recategorized into two groups, white and other ethnicities. Obtainable education (UKB Field 6138-0.0) was categorized into the College/University degree and above or below College/University degree. Deprivation was determined by Townsend Index (UKB Field 189-0.0), which is a measure of material deprivation of participants' postcode location area based on the preceding national census output area taking into account unemployment, non-car ownership, non-home ownership, and household crowding. Smoking status (UKB Field 20116-0.0) was categorized into never smokers and ex/current smokers. The physical activity (UKB Field 22036-0.0) was defined according to whether one met the 2017 UK Physical activity guidelines of 150 min of walking or moderate activity per week or 75 min of vigorous activity.

Hypertension was defined as having blood pressure treatment, a self-reported hypertension history, systolic blood pressure ≥ 130 mm Hg, or diastolic blood pressure ≥ 80 mm Hg; and diabetes mellitus was defined as previous diabetes diagnosed by a doctor, the use of insulin or other diabetes-related medication, or HbA1c over 48 mmol/mol; depression was either self-reported prior depression diagnosis or PHQ-2 depression score ≥ 3. Self-reported overall health status (UKB Field 2178-0.0) were categorized into excellent/good or fair/poor. Identification of ApoE e4 allele was based on genome data provided by UKB.

### Statistical Analysis

Continuous variables (e.g., age) were reported as means and standard deviations and compared using unpaired *t*-test. Categorical variables were reported as numbers and percentages and compared through Pearson's chi-square test. Cox proportional hazards model was used to identify the risks of incident dementia associated with VI, HI, and DSI, adjusting for age and gender in model 1, or for age, gender, ethnicity, Townsend index, obtainable education, physical activity, history of hypertension, diabetes, and depression and overall health status in model 2. Schoenfeld Residuals were used to test the proportional-hazards assumption. Risks were shown in hazard ratio (*HR*) with 95% confidence interval (*CI*). Sensitivity analysis was performed by excluding incidence cases of dementia within 1 year of the baseline assessment, to minimize the potential effect of undiagnosed dementia. All *p-*values were two-sided, and a *p* < 0.05 was considered statistically significant. Analyses were performed using Stata version 14 (version 14.0; StataCorp).

## Results

### Study Sample

A total number of 113,511 participants without dementia at baseline were included in the present study, with a mean age of 56.8 ± 8.09 years, a range of age from 39 to 72 years, and 54.4% women. The baseline characteristics stratified by the status of sensory impairments were shown in Table 1. There were significant differences amongst the groups in terms of age, gender, ethnicity, Townsend index, obtainable education, physical activity, history of hypertension, diabetes, and depression and overall health status.

**Table 1:** Baseline characteristics of participants stratified by the status of sensory impairment.

**Baseline characteristics**	**Total**	**NSI**	**VI-only**	**HI-only**	**DSI**	***p***
*N*	113,511	96,265	2,969	13,547	730	
Age, mean (SD), years	56.8 (8.09)	56.3 (8.08)	58.3 (7.60)	59.9 (7.51)	60.9 (6.87)	**<0.001**
Gender, *N* (%)						**0.025**
Female	61,831 (54.5)	52,566 (54.6)	1,647 (55.5)	7,221 (53.3)	397 (54.4)	
Male	51,680 (45.5)	43,699 (45.4)	1,322 (44.5)	6,326 (46.7)	333 (45.6)	
Ethnicity, *N* (%)						**<0.001**
White	102,090 (89.9)	88,253 (91.7)	2,616 (88.1)	10,677 (78.8)	544 (74.5)	
Others	11,421 (10.1)	8,012 (8.32)	353 (11.9)	2,870 (21.2)	186 (25.5)	
Townsend Index, mean (SD)	−0.99(2.98)	−1.11 (2.92)	−0.47 (3.21)	−0.30 (3.22)	0.47 (3.43)	**<0.001**
Education, *N* (%)						**<0.001**
College/University degree	39,847 (35.1)	35,110 (36.5)	869 (29.3)	3,722 (27.5)	146 (20.0)	
Without College/University degree	73,664 (64.9)	61,155 (63.5)	2,100 (70.7)	9,825 (72.5)	584 (80.0)	
Smoking status, *N* (%)						0.148
Never	62,651 (55.4)	53,260 (55.5)	1,644 (55.6)	7,369 (54.8)	378 (52.4)	
Prior/current	50,456 (44.6)	42,712 (44.5)	1,312 (44.4)	6,088 (45.2)	344 (47.6)	
Above moderate/vigorous/walking recommendation, *N* (%)						**0.040**
No	16,381 (17.6)	13,987 (17.6)	404 (16.9)	1,868 (17.8)	122 (21.9)	
Yes	76,484 (82.4)	65,451 (82.4)	1,992 (83.1)	8,606 (82.2)	435 (78.1)	
History of hypertension, *N* (%)						**<0.001**
No	29,184 (25.7)	25,718 (26.7)	656 (22.1)	2,693 (19.9)	117 (16.0)	
Yes	84,327 (74.3)	70,547 (73.3)	2,313 (77.9)	10,854 (80.1)	613 (84.0)	
History of diabetes mellitus, *N* (%)						**<0.001**
No	106,173 (93.5)	90,700 (94.2)	2,747 (92.5)	12,095 (89.3)	631 (86.4)	
Yes	7,338 (6.46)	5,565 (5.78)	222 (7.48)	1,452 (10.7)	99 (13.6)	
History of depression, *N* (%)						**<0.001**
No	102,076 (89.9)	87,098 (90.5)	2,649 (89.2)	11,738 (86.7)	591 (81.0)	
Yes	11,435 (10.1)	9,167 (9.52)	320 (10.8)	1,809 (13.3)	139 (19.0)	
Self-rated health status, *N* (%)						**<0.001**
Excellent/good	81,828 (72.4)	70,708 (73.7)	2,016 (68.3)	8,677 (64.6)	427 (59.1)	
Fair/poor	31,247 (27.6)	25,252 (26.3)	935 (31.7)	4,764 (35.4)	296 (40.9)	
APOE e4, *N* (%)						0.266
Absent	83,242 (75.9)	70,684 (75.9)	2,211 (77.1)	9,825 (75.6)	522 (74.5)	
Present	26,436 (24.1)	22,423 (24.1)	656 (22.9)	3,178 (24.4)	179 (25.5)	

*NSI, neither sensory impairment; VI, visual impairment; HI, hearing impairment; DSI, dual-sensory impairment; SD, standard deviation*.

*Bold values suggest a P value < 0.05*.

### Incidence of Dementia

During the mean follow-up period of 11.1 years (*IQR*: 10.9–11.4 years), a total of 1,135 (1.00%) participants developed all-cause dementia. Of these incident cases, 789 were in the NSI group, 42 in the VI-only group, 283 in the HI-only group, and 21 in the DSI group. The log-rank test demonstrated a significant difference in the incidence of dementia among the four groups (*p* < 0.001). Table 2 demonstrated the baseline characteristics of dementia and non-dementia group. As shown in the age- and gender-adjusted Cox proportional hazard regression models, age, male gender, increased level of deprivation, less education, smoking, lack of physical activity, history of hypertension, diabetes and depression, poor overall health status, and the presence of APOE e4 allele were risk factors for developing dementia.

**Table 2:** Baseline characteristics of participants stratified by incident dementia.

**Baseline characteristics**	**Non-dementia**	**Incident dementia**	***p***	**HR (95% *CI*)^**a**^**	***p***
*N*	112,376	1,135			
Age, mean (SD), years	56.7 (8.08)	64.0 (5.11)	**<0.001**	1.18 (1.17–1.20)	**<0.001**
Gender, *N* (%)			**<0.001**		
Female	61,308 (54.6)	523 (46.1)		1 [Reference]	
Male	51,068 (45.4)	612 (53.9)		1.31 (1.16–1.47)	**<0.001**
Ethnicity, *N* (%)			**0.004**		
White	101,040 (89.9)	1,050 (92.5)		1 [Reference]	
Others	11,336 (10.1)	85 (7.49)		1.16 (0.93–1.44)	0.195
Townsend Index, mean (SD)	−0.99 (2.98)	−0.67 (3.17)	**<0.001**	1.07 (1.05–1.10)	**<0.001**
Education, *N* (%)			**<0.001**		
College/University degree	39,597 (35.2)	250 (22.0)		1 [Reference]	
Without College/University degree	72,779 (64.8)	885 (78.0)		1.53 (1.33–1.76)	**<0.001**
Smoking status, *N* (%)			**<0.001**		
Never	62,116 (55.5)	535 (47.4)		1 [Reference]	
Prior/current	49,862 (44.5)	594 (52.6)		1.16 (1.03–1.30)	**0.016**
Above moderate/vigorous/walking recommendation, *N* (%)			0.169		
No	16,214 (17.6)	167 (19.4)		1 [Reference]	
Yes	75,791 (82.4)	693 (80.6)		0.78 (0.65–0.92)	**0.003**
History of hypertension, *N* (%)			**<0.001**		
No	29,027 (25.8)	157 (13.8)		1 [Reference]	
Yes	83,349 (74.2)	978 (86.2)		1.23 (1.04–1.46)	**0.018**
History of diabetes mellitus, *N* (%)			**<0.001**		
No	105,225 (93.6)	948 (83.5)		1 [Reference]	
Yes	7,151 (6.36)	187 (16.5)		2.21 (1.89–2.59)	**<0.001**
History of depression, *N* (%)			**<0.001**		
No	101,115 (90.0)	961 (84.7)		1 [Reference]	
Yes	11,261 (10.0)	174 (15.3)		2.22 (1.88–2.61)	**<0.001**
Self-rated health status, *N* (%)			**<0.001**		
Excellent/good	81,210 (72.5)	618 (54.8)		1 [Reference]	
Fair/poor	30,737 (27.5)	510 (45.2)		2.29 (2.03–2.57)	**<0.001**
APOE e4, *N* (%)			**<0.001**		
Absent	82,643 (76.1)	599 (54.9)		1 [Reference]	
Present	25,944 (23.9)	492 (45.1)		2.69 (2.39–3.03)	**<0.001**

*SD, standard deviation; HR, hazard ratio; CI, confidence interval*.

a *Adjusted for age and gender*.

*Bold values suggest a P value < 0.05*.

### Sensory Impairments and Incident Dementia

Age- and gender-adjusted Cox proportional hazard regression models (Table 3) showed that participants with VI, HI, and DSI had a 1.42-fold (95% *CI*: 1.04–1.94, *p* = 0.026), 1.71-fold (95% *CI*: 1.49–1.96, *p* < 0.001), and 2.23-fold (95% *CI*: 1.44–3.44, *p* < 0.001) increased risk of developing dementia, respectively, compared with those with neither sensory impairment. In the multivariable-adjusted models, isolated VI (*HR* = 1.50, 95% *CI*: 1.06–2.12, *p* = 0.023), isolated HI (*HR* = 1.42, 95% *CI*:1.20–1.69, *p* < 0.001), and DSI (*HR* = 1.82, 95% *CI*: 1.10–3.00, *p* = 0.020) were independently associated with increased risk of incident dementia (Table 3).

**Table 3:** Association of sensory impairment status with incident dementia.

**Sensory**	**Age- and**		**Multivariable**	
**impairment status**	**gender-adjusted model**		**model^a^**	
	**HR (95% CI)**	***P***	**HR (95% CI)**	***P***
**Main analysis**				
NSI	1[Reference]		1[Reference]	
VI-only	1.42 (1.04–1.94)	**0.026**	1.50 (1.06–2.12)	**0.023**
HI-only	1.71 (1.49–1.96)	**<0.001**	1.42 (1.20–1.69)	**<0.001**
DSI	2.23 (1.44–3.44)	**<0.001**	1.82 (1.10–3.00)	**0.020**
**Sensitivity analysis^b^**				
NSI	1[Reference]		1[Reference]	
VI-only	1.43 (1.05–1.95)	**0.023**	1.50 (1.06–2.12)	**0.022**
HI-only	1.71 (1.49–1.96)	**<0.001**	1.42 (1.20–1.68)	**<0.001**
DSI	2.24 (1.45–3.46)	**<0.001**	1.82 (1.10–3.01)	**0.019**

*NSI, neither sensory impairment; VI, visual impairment; HI, hearing impairment; DSI, dual-sensory impairment; HR, hazard ratio; CI, confidence interval*.

a *Cox proportional hazards regression models adjusted for age, gender, ethnicity, education, townsend index, smoking status, physical activity, history of hypertension, diabetes and depression, self-rated health status and APOE e4 allele*.

b *Participants with incident dementia diagnosed within 1 year from the baseline assessment were excluded*.

*Bold values suggest a P value <0.05*.

### Sensitivity Analysis

The results of the sensitivity analysis were shown in Table 3. After excluding participants who were diagnosed with all-cause dementia within 1 year of the baseline assessment, the associations found in the main analysis (both the age- and gender-adjusted model and the multivariable-adjusted model) remained significant.

## Discussion

In this large prospective cohort study of 113,511 individuals, we found that participants with isolated VI, isolated HI and DSI had 50, 42, and 82% higher risk, respectively, of developing dementia compared to those with neither sensory impairment, indicating that sensory impairments are potential interventional targets for dementia.

Our findings added important evidence to the body of knowledge on the relationship of VI, HI and DSI with dementia using objective measurements of VA and SRT. The impact of DSI on dementia found in the present study was consistent with previous cross-sectional and longitudinal evidence (Yamada et al., 2016; Luo et al., 2018; Michalowsky et al., 2019; Hwang et al., 2020; Maharani et al., 2020; Byeon et al., 2021; Kuo et al., 2021). However, Hong et al. reported neither isolated VI/HI nor DSI was associated with cognitive decline in the Blue Mountains Eye Study (Hong et al., 2016). This may be partly explained by the ascertainment of VI and HI based on the worse eye and ear, which did not take into account the functional compensation by the better eye or ear.

Furthermore, the relationship between isolated sensory impairment and dementia was inconclusive. Several cross-sectional and prospective studies have shown that VI but not HI was independently associated with dementia (Luo et al., 2018; Hwang et al., 2020). In contrast, other studies reported that HI but not VI, or neither sensory impairment alone, was associated with increased risks of incident dementia (Michalowsky et al., 2019; Byeon et al., 2021). To sum up, heterogeneities in study design, demographic factors of the participants, the definition of sensory impairments, ascertainment of dementia, and adjustment of covariates could account for these discrepancies. More importantly, adoption of whether subjective or objective approaches to define sensory impairment could contribute to the inconclusive findings. Of note, prevalence of VI (Whillans and Nazroo, 2012) and HI (Kiely et al., 2012) based on self-reports were overestimated compared with standardized objective measures. Although it is difficult to define whether objective measurements dwarf subjective approaches in clinical value, objective methods have incremental benefit in generating more conservative conclusions.

Several mechanisms may explain the associations found in the present study. The first hypothesis is the “common cause” theory (Valentijn et al., 2005), which states the common pathology and risk factors shared between dementia and sensory impairment may result in their strong associations. Mounting evidence suggested that the hallmark pathological features of dementia in the brain, such as A-beta plaques, pTau, and vascular alterations, affects the peripheral sensory organs (i.e., retina and cochlea), and the visual and auditory pathways as well (Leuba and Saini, 1995; Baloyannis et al., 2009; Koronyo-Hamaoui et al., 2011; La Morgia et al., 2016; Omata et al., 2016). Second, sensory impairment and cognitive dysfunction shared common risk factors such as diabetes, hypertension, obesity, and smoking (Shang et al., 2021). Third, the reduced information input secondary to sensory impairment could result in reduced stimulation of the cognitive domains of the brain (Griffiths et al., 2020). Evidence from recent studies found decreased volumes of specific brain regions involved in visual processing, and atrophy of total brain volume and the limbic system implicated in neurodegenerative diseases in visually impaired individuals (Bathelt et al., 2020; Zhu et al., 2021). Fourth, sensory impairment could contribute to the development of dementia directly or indirectly mediated by depression, one of the most well-established risk factors for dementia (Jorm, 2000; Cacioppo and Hawkley, 2009; Steenland et al., 2012). Lastly, sensory impairment may lead to increased cognitive load driven by the engagement of more cognitive resources for visual and acoustic tasks, leaving limited resources available for cognitive tasks such as working memory, speech, and attention (Griffiths et al., 2020).

Our findings demonstrated public health implications in dementia prevention. The positive association of VI, HI, and DSI with incident dementia highlighted the importance of sensory preservation and rehabilitation to prevent or slow down the progression of dementia. Vision impairment and hearing loss were estimated to contribute to 4.7 and 8.2% of all cases of dementia, respectively (Livingston et al., 2020; Shang et al., 2021). Notably, ~80% causes of VI and 60% causes of HI are avoidable or treatable (Pascolini and Mariotti, 2012; Organization, 2016). The majority of them could be intervened by currently available and cost-effective measures such as screening programs, vaccination, cataract surgery, refractive spectacles, and hearing aids (Blindness et al., 2021; GBD Hearing Loss Collaborators, 2021). In addition, growing evidence suggested that preventive or treatment measures for the leading causes of VI and HI were associated with improved cognitive function (Tamura et al., 2004; Ishii et al., 2008; Dawes et al., 2015; Sarant et al., 2020). Therefore, the important role of screening and treatment for sensory impairment in reducing the global burden of dementia should be emphasized.

There are several strengths of this study, including the large sample scale, long follow-up period, objective measurement of sensory impairments, and comprehensive adjustment for confounding factors. There are also several limitations. First of all, the relatively low incidence of dementia in the cohort prevented us from performing subtype analyses of specific types of dementia. Second, because of the lack of data on the exact causes of VI and HI, we could not investigate the association of specific ocular or acoustic disorders with the risk of developing dementia. Third, the UK biobank study might have “health selection bias” due to its relatively young and healthy groups of participants. Although this should not affect the association between the exposure and the outcome (Fry et al., 2017). Fourth, the UK Biobank algorithm were only able to recognize clinically significant cases of dementia, which may reduce the diagnostic accuracy. Last, the possibility of residual confounding could not be excluded given the observational nature of our study even if we have adjusted for a variety of conventional confounding factors of dementia.

## Conclusion

In conclusion, in this large population-based prospective cohort study, we found VI, HI, and DSI were associated with significantly increased risk of developing dementia, indicating that sensory impairments are important modifiable risk factors for dementia. Preventative or therapeutic interventions for VI and HI should be made widely available and their significance beyond the scope of improving sensory functions should be highlighted.

## Data Availability Statement

Publicly available datasets were analyzed in this study. This data can be found here: https://www.ukbiobank.ac.uk. Access to the data was granted through application and the application number for the present study is 62525.

## Ethics Statement

The studies involving human participants were reviewed and approved by North West Multi-Centre Research Ethics Committee (11/NW/0382). The patients/participants provided their written informed consent to participate in this study.

## Author Contributions

WH, WW, YW, ZZ, XY, and MH: study concept and design. WH and XiZ: drafting of the manuscript. WW, YW, YC, ZZ, XY, and MH: critical revision of the manuscript for important intellectual content. WH and ZZ: statistical analyses. XY and MH: obtained funding. WW, XS, ZZ, XY, and MH: administrative, technical, or material support. ZZ, XY, and MH: study supervision. All authors: acquisition, analyses, or interpretation. All authors contributed to the article and approved the submitted version.

## Funding

The present work was supported by Fundamental Research Funds of the State Key Laboratory of Ophthalmology, National Natural Science Foundation of China (82000901, 82101173, 81870663, 82171075), NHMRC Investigator Grants (2010072), Outstanding Young Talent Trainee Program of Guangdong Provincial People's Hospital (KJ012019087), Guangdong Provincial People's Hospital Scientific Research Funds for Leading Medical Talents and Distinguished Young Scholars in Guangdong Province (KJ012019457), Talent Introduction Fund of Guangdong Provincial People's Hospital (Y012018145), Science and Technology Program of Guangzhou, China (202002020049), Project of Special Research on Cardiovascular Diseases (2020XXG007), and Research Foundation of Medical Science and Technology of Guangdong Province (B2021237). MH receives support from the University of Melbourne at Research Accelerator Program and the CERA Foundation. The Centre for Eye Research Australia receives Operational Infrastructure Support from the Victorian State Government. The sponsor or funding organization had no role in the design or conduct of this research.

## Conflict of Interest

The authors declare that the research was conducted in the absence of any commercial or financial relationships that could be construed as a potential conflict of interest.

## Publisher's Note

All claims expressed in this article are solely those of the authors and do not necessarily represent those of their affiliated organizations, or those of the publisher, the editors and the reviewers. Any product that may be evaluated in this article, or claim that may be made by its manufacturer, is not guaranteed or endorsed by the publisher.

## Abbreviations

VI: visual impairment
HI: hearing impairment
DSI: dual sensory impairment
NSI: non-sensory impairment
VA: visual acuity
LogMAR: logarithm of the minimum angle of resolution
SRT: speech-reception-threshold
ICD: International Classification of Diseases
NHS: National Health Service
HR: hazard ratio
CI: confidence interval.
