# Supplementary material for: Association of Visual, Hearing, and Dual Sensory Impairment With Incident Dementia
Source: Front Aging Neurosci. 2022 Jun 14;14:872967. doi: 10.3389/fnagi.2022.872967 (PMC9239339; doi:10.3389/fnagi.2022.872967)
Supplement: Supplementary file 1 [file Table_1.pdf]

Supplementary Table1. Baseline characteristics of participants with and without VA and (or) SRT data

Baseline characteristics	Participants without VA and (or) SRT data	Participants with VA and SRT data	P value
N	388,929	113,563	
Age, mean(SD), years	56.4 (8.09)	56.8 (8.09)	**<0.001**
Gender, N(%)			0.655
Female	211,528 (54.4)	61,849 (54.5)	
Male	177,401 (45.6)	51,714 (45.5)	
Ethnicity, N(%)			**<0.001**
White	370,544 (95.3)	102,139 (89.9)	
Others	18,386 (4.73)	11,424 (10.1)	
Townsend Index, mean(SD)	-1.38 (3.12)	-0.99 (2.98)	**<0.001**
Education, N(%)			**<0.001**
College/University degree	121,295 (31.2)	39,866 (35.1)	
Without College/University degree	267,635 (68.8)	73,697 (64.9)	
Smoking status, N(%)			**<0.001**
Never	210,845 (54.6)	62,671 (55.4)	
Prior/current	175,540 (45.4)	50,488 (44.6)	
Above moderate/vigorous/walking recommendation, N(%)			**<0.001**
No	58,203 (18.8)	16,386 (17.6)	
Yes	251,154 (81.2)	76,516 (82.4)	
History of hypertension, N(%)			**<0.001**
No	109,988 (28.3)	29,195 (25.7)	
Yes	278,942 (71.7)	84,368 (74.3)	

History of diabetes mellitus, N(%)			**<0.001**
No	366,009 (94.1)	106,223 (93.5)	
Yes	22,921 (5.89)	7,340 (6.46)	
History of depression, N(%)			**0.005**
No	350,811 (90.2)	102,114 (89.9)	
Yes	38,119 (9.80)	11,449 (10.1)	
Self-rated health status, N(%)			**<0.001**
Excellent/good	289,013 (74.9)	81,853 (72.4)	
Fair/poor	96,865 (25.1)	31,274 (27.6)	
APOE e4, N(%)			0.749
Absent	287,096 (78.9)	83,277 (75.9)	
Present	91,417 (24.1)	26,449 (24.1)	

Abbreviations: VA=visual acuity, SRT=speech-reception threshold, SD=standard deviation
